# Removing barriers to participation in clinical trials, a conceptual framework and retrospective chart review study

**DOI:** 10.1186/1745-6215-13-237

**Published:** 2012-12-10

**Authors:** Norma F Kanarek, Marty S Kanarek, Dare Olatoye, Michael A Carducci

**Affiliations:** 1 Department of Environmental Health Sciences, Johns Hopkins Bloomberg School of Public Health, 615 North Wolfe Street, Baltimore, MD 21205, USA; 2 Department of Oncology, Johns Hopkins School of Medicine, 615 North Wolfe Street, Baltimore, MD 21205, USA; 3 Department of Population Health Sciences, School of Medicine and Public Health and the Nelson Institute for Environmental Studies, University of Wisconsin-Madison, 610 North Walnut Street, Madison, WI, 53726, USA; 4 Johns Hopkins School of Medicine, 733 North Broadway, Baltimore, MD, 21205-2196, USA; 5 Department of Oncology (Urologic Oncology), Johns Hopkins School of Medicine, Cancer Research Building I, Room 1M59, Baltimore, MA, USA

**Keywords:** Justice, Beneficence, Respect for persons, Clinical trials, Clinical trial accrual, Prostate cancer, Barriers to clinical trials, Disparities, Framework

## Abstract

**Background:**

Enrollment in interventional therapeutic clinical trials is a small fraction of all patients who might participate given reasonable access.

**Methods:**

A hierarchical approach is utilized in measuring staged participation from trial availability to patient enrollment. Our framework suggests that concern for justice comes in the design and eligibility criteria for clinical trials; attention to beneficence is given in the eligibility and physician triage stages. The remaining four stages rely on respect for persons. An example is given where reasons for nonparticipation or barriers to participation in prostate cancer clinical trials are examined within the framework. In addition, medical oncology patients with an initial six month consultation are tracked from one stage to the next by race using the framework to assess participation comparability.

**Results:**

We illustrated seven transitions from being a patient to enrollment in a clinical trial in a small study of prostate cancer cases who consulted SKCCC Medical Oncology Department in early 2010. Pilot data suggest transition probabilities as follows: 65% availability, 84% eligibility, 92% patient triage, 89% trials discussed, 45% patient interested, 63% patient consented, and 92% patient enrolled. The average transition probability was 77.7%. The average transition probability, patient-trial-fit was 50%; opportunity was 51%, and acceptance was 66.7%. Trial availability, patient interest and patient consented were three transitions that were below the average; none were statistically significant.

**Conclusions:**

The framework may serve to streamline comprehensive reporting of clinical trial participation to the benefit of patients and the ethical conduct of clinical trials.

## Background

Enrollment in interventional therapeutic clinical trials involves a small fraction of all patients who might participate given reasonable access. On examining patients with incident cancer in the USA [1], California, USA [2] and Maryland, USA [3], less than 3% participated in available trials [4]. Moreover, patients who are seen at a cancer center, are representative of neither the population nor cancer cases [4,5]. A large proportion of government-sponsored trials are conducted at National Cancer Institute (NCI) cancer centers [1], located such that 45% of all Americans live within a reasonable travel time [6], and among patients who attend these cancer centers, a higher proportion of cancer patients participate [2,7]. Once seen at a cancer center, the proportion of those for whom the physician would recommend a trial, for whom a trial is available, and for whom trial eligibility criteria are met is about 25% [8]. Nevertheless, enrollment may be improved as we learn more about barriers to participation.

A number of factors influence enrollment in clinical trials. Prior research has reported system, institution, physician, and patient domains that merit attention when trying to improve participation [9,10]. These same domains may differentially affect access to clinical trials for subpopulations [11,12]. Institutional investigations may attempt to improve specific trial accrual, accrual across all populations and cancer sites, or for specific patient groups [13,14].

A scan of the literature has identified several stages of accrual to clinical trials. These stages include: trial availability [15,16], study eligibility [17], physician triage (preference or judgment about care outside the parameters of the trial requirements) [15,16,18], presentation of the trial(s) [18], determination of patient interest and barriers [15,19], and acquisition of informed consent and enrollment [13]. In the same literature sources, the ordering of stages differs, or stages are combined. For instance, some investigators engage physician triage first and then determine if the patient’s condition fits any available trial. In another case, trial availability and eligibility are conflated [8]. To date, a hierarchical approach with transitional probabilities is universally used though the order of accrual stage differs. Meanwhile, reporting differences make comparisons difficult.

The ethical conduct of research in human subjects, including the process of recruitment to clinical trials, is the responsibility of trial principal investigator(s) (PIs) and Institutional Review Boards (IRBs). While PIs are ultimately responsible for how they conduct their research, local IRBs are responsible for review and oversight of all human subject research conducted at their institution. The ethical review conducted by IRBs is guided by three principles: respect for persons, beneficence and justice [19]. In practice, these principles focus the attention of the IRB on assuring that the proposed trial will further knowledge, be methodologically sound, have fair selection practices, possess a favorable risk-benefit ratio, be judged by those independent of the study team, include information on how the investigators plan to obtain informed consent, and assure mechanisms are in place respect individuals and their privacy [20].

In this paper we propose an ordered and comprehensive set of accrual stages that are supported by ethical considerations and comprise concepts reported in the literature to date. These steps are illustrated with data from a chart review of new prostate cancer cases seen in medical oncology for six months.

## Methods

Prostate cancer patients seen by three senior medical oncologists for a first visit between January and April 2010 were studied (n = 94). Patient information was gleaned from patient records and supplemented by the Johns Hopkins Hospital Cancer Registry. Any clinical trial participation during the subsequent six months was abstracted. These 94 prostate cancer cases represent 68% of all new Genito-Urinary Program consultations in medical oncology during this period. Overall patient enrollment is the number of enrollees divided by all patients presenting. Race-specific enrollment is delimited by specific race or ethnicity.

Transitional probabilities (percentage in the current step of those in the prior step) were calculated. To summarize all transition probabilities, we calculated the overall seven-step transitional probability by taking the seventh root of the overall enrollment percentage of all patients. Three multistep transition probabilities were also calculated (multiplicands of component transitional probabilities).

The chi square test for independence was used to evaluate demographics of those eligible/not eligible in the case series and those enrolled/not enrolled; none reached statistical significance in this pilot and small sample size series.

The Clinical Research Office at the Sidney Kimmel Comprehensive Cancer Center (SKCCC) reviewed and approved the study.

### Patient to accrual framework

As noted earlier, ethical clinical trial enrollment is made up of 1) scientific oversight, 2) trial availability, 3) patient eligibility, 4) physician triage, 5) discussion of trials, 6) ascertainment of patient interest and willingness to participate in a particular trial, 7) consent for participation, and 8) enrollment. Each of these eight important steps is implicitly or explicitly a consideration in enrollment of patients into clinical trials. We suggest that there is an ethical hierarchy, that is, each step must be fulfilled before proceeding to the next step to fulfill obligations to provide trials embodying justice, beneficence, and autonomy [19].

#### Scientific oversight

Scientific oversight is the responsibility and the domain of the institution. Trials are designed, reviewed, approved and joined with the goal of gaining generalizable knowledge about whether treatment is better than current modalities, and as proposed, the study has benefits to future patients and may have benefits to those in the trial (beneficence). Impediments to trial participation at this stage are that the institution cannot identify patients who will benefit, that patients do not attend the institutions where the trials are offered, or that the institution cannot launch trials efficiently. This strikes at the heart of clinical trial performance - failure to accrue [21]. Enrollment of sufficient subjects is often the basis for multi-center trials when no one institution can possibly enroll enough patients in the optimal time window. Hereafter, we assume at the first step that SKCCC is offering trials that provide generalizable knowledge using rigorous methods and monitored by independent scientific oversight (Table 1).

**Table 1:** Reasons for not participating in a therapeutic clinical trials by domain and ethical factors

	**Domain**	**Ethical requirements**[19]	**Ethical principles**[18]	**Sample reasons for not participating in a clinical trial**[15,22,41,52]
Patient-trial fit				
Scientific oversight [10,53]	Institution	5-Independent review	Beneficence	Cannot identify patients
				Patients seek services elsewhere
		2-Scientific validity		Cannot enroll enough patients
				Unavailability
				No measureable/confirmable disease
Availability by disease characteristics [1,20]		1-Social value	Justice	No testable treatments for the disease, stage, or other characteristics
	Investigators			
Eligibility [9]		3-Fair subject selection justice		Prior tumor, second cancer, metastases
				Precluded by prior or current treatment or co-morbid conditions
			Beneficence	Poor performance status
				Asymptomatic
				Study demands
Physician triage [25]	Physician	4-Favorable risk-benefit ratio		Treatment not tolerated, died soon, disease is too advanced
				Quality of life issues
				Life expectancy is limited
				Need for immediate treatment
				Physician preference/judgment
Opportunity				
Discussion of trials [27,33,36,37]	Physician	6-Informed and voluntary consent	Respect for persons	Physician judged compliance issues
				Doctor-patient communication issues
Interest and willingness [24,29,30,39]	Patient	6-Informed and voluntary consent	Respect for persons	Treatment preference /placebo
				Minimal care: symptomatic treatment only, supportive or hospice care desired, refused further staging
				Not interested
				Barriers: distant from clinic [5], financial, insurance [48], personal circumstances
				Second opinion only, return to home physician, no ongoing relationship
Acceptance				
Patient consent [45]	Patient	6-Informed and voluntary consent	Respect for persons	Fear of randomization
				Fear of side effects
				Negative aspects of a trial participation, distance
Patient enrollment	Investigator	7-Respect for enrolled subjects		Patient failed trial screening protocol
				Withdrawal

#### Trial availability by disease characteristics

Trial availability by disease characteristics is both an institutional and an investigator domain. Availability of a trial is defined as: a trial exists that is open, actively enrolling patients, and appropriate to the patient’s condition (for example, cancer site and stage [8]) or patient class [22,23]. Broadly, availability measures the ability to serve a significant proportion of the patients who enter that research institution and ethically measures fair distribution (justice) of risks, benefits and costs. From prior reports, trial availability is usually the primary barrier to participation in clinical trials [15,16].

#### Eligibility

Eligibility is an investigator domain and is managed in trial design. Eligibility for a trial is determined by the actual requirements of individual trials and includes study-defined patient determinants (for example, risk [12], prior treatment [24]) or a person’s ability to meet the participation requirements of the trial (for example, follow-up attendance). To avoid doing harm (beneficence) and to implement fair subject selection criteria (justice), eligibility is established for each trial. In the case of early-phase trials, criteria are designed to predict tolerance of the maximum dose and are often characterized by comorbidities, biologic measures of functioning (for example, red blood count), drug tolerance, prognosis, and with judiciousness, by patient demographics [25-27].

#### Physician triage

Physician triage [26] is the domain of the patient’s doctor. The treating and consulting physicians, whether it is the primary-care provider or cancer specialist, makes decisions about patient suitability for clinical trials in order to minimize harm. A patient who is inaccurately assessed as eligible could be harmed by enrollment (lack of beneficence). Physicians who know the patient can make decisions about whether for particular patients a reasonable risk-benefit balance is not met as judged by quality of life and life expectancy information, whether the patient requires immediate treatment, or whether deference for a person’s last days, or their decision to treat no further should hold sway. Physician presumption about the patient’s ability or willingness to participate (for example, based on age or distance from the clinic) is sometimes reported in this category [26]. While physicians may have knowledge about these potentially real barriers to participation in trials, we suggest physician presumptions should be assiduously avoided at this stage [27]. This step involves a therapeutic orientation, which is concern for the person as patient in contrast to study subject [28].

#### Patient-trial fit

Patient-trial fit is the percentage of all patients for whom there is a trial available, eligibility requirements are met, and the physician has deemed the patient a trial candidate. Patient-trial fit is in the domains of institution, investigators, and physician and is a comprehensive measure of the match of the institutional trial portfolio, and its clientele. The ethics of justice and beneficence are demonstrated here.

#### Discussion of trials

Discussion of trials between the treating physician and the patient is most often in the physician domain to initiate [29-31], though more and more frequently patients raise the possibility with their doctors, the patient domain [32,33], and this is increasingly recognized as physician-patient partnership [34,35]. Discussion of trials occurs to ascertain whether a patient is able to understand the nature of the clinical trial [36,37] and its benefits and costs, and to assess the patient’s ability to provide informed consent [38]. It is also a significant recognition that patient autonomy is a value that to act upon entails allowing each patient to decide about trial participation to the extent of their ability. In the past and especially for community physicians, convenience, cost, and necessary follow-up visits in addition to age were perceived as barriers for their patients and so trials were not discussed [13,39]. Go and colleagues [15] describe as reality that about a quarter of community physicians think risks outweigh benefits at this stage and do not discuss trials with their patients - a clear and relevant factor in physician triage. Discussion of a trial with every patient for whom there is patient-trial fit, however, respects each person and their decision making.

#### Patient interest and willingness

Patient interest and willingness in participation in a clinical trial is in the patient domain (including family members and caregivers). The majority of cancer patients are unaware that they might be eligible for a clinical trial, or that trials are conducted where they are receiving treatment [10]. Interest in a trial is predicated on respect for persons and may be associated with optimism [40], gender [41], more information (cancer site, age > 80 years, serious disease status) [13,32,33,38], type of intervention, marital status, race [42], physician communication styles [43], and other factors [27].

#### Opportunity

Opportunity is the percentage of those patients who fit the criteria for trial inclusion, who are interested in and willing to participate in a clinical trial. Opportunity is in the domains of both the physician and patient and is an aggregate measure of providing the option of trial participation among those for whom there is a trial that fits their characteristics and embodies the ethic of respect for persons.

#### Patient consent

Patient consent is a domain stage of clinical trial accrual and is linked closely to respect for patient autonomy [34,44]. Issues related to prostate cancer participation in clinical trials that facilitate consent include patient preferences for specific interventions [45], lower socioeconomic status [46], cost of travel and friends/family to accompany the patient [43,47], availability of the intervention outside a trial setting [48], and an intervention that ‘kills cancer cells’ [49]. Under some circumstances, parents, guardians, or family may have an important consent or support role. In the study of other diseases additional factors may be found to affect patient consent.

#### Patient enrollment

Patient enrollment is in the study investigator domain and assesses whether the patient meets the specific study requirements at the time of enrollment, and then begins care in the clinical trial. Impediments at this point may include lack of insurance coverage [8,18,50], staff support, and timely participation slots.

#### Acceptance

Acceptance is the percentage of interested and willing patients with whom a trial was discussed, who accept a clinical trial, and are accepted into a clinical trial. Acceptance is a measure of patient enrollment once trial-patient fit and opportunity are confirmed.

## Results

**Figure 1:**
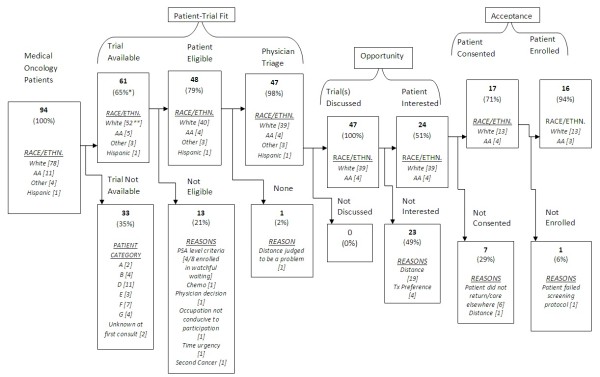
Flow chart of prostate cancer patient enrollment in medical oncology clinical trials, January to April 2010, Sidney Kimmel Comprehensive Cancer Center.

We illustrated seven transitions from being a patient to enrollment in a clinical trial in a small pilot study of about a hundred prostate cancer cases who consulted SKCCC Medical Oncology Department in early 2010. Figure 1 illustrates accrual stages 2 to 8 in a sample of prostate cancer patients seen for a first visit by three experienced medical oncologists and followed for 6 months. At each stage of accrual, patients are categorized from trial availability to enrollment. Percentages in parentheses are transitional probabilities based on the number of patients available at the previous stage. In Figure 1, we illustrate the reasons given for not continuing toward enrollment at each stage.

### Clinical trial enrollment

Of the 94 patients seeking care, 11 were enrolled in medical oncology clinical trials. The white enrollment rate was 10% (8/78), black enrollment was 27% (3/11), and enrollment among other races and Hispanic ethnicity was 0% (0/5).

### Transition probabilities

We obtained transitional probabilities as follows: 65% (61/94) availability, 84% (51/61) eligibility, 92% (47/51) physician triage, 89% (42/47) trials discussed, 45% (19/42) patient interested, 63% (12/19) patient consented, and 92% (11/12) patient enrolled. The average transitional probability was 74%. The aggregate transitional probability, patient-trial fit was 50% (47/94); opportunity was 40% (19/47), and acceptance was 58% (11/19). Trial availability, patient interested and patient consented were three transitions that were below average; however, none was statistically significant.

### Reasons for drop-off

We categorized drop-off from stage to stage. Of the thirty-three patients who were not eligible for a trial, six (18%) had pre-recurrent disease, fourteen (42%) had recurrent prostate cancer, and eleven (33%) had metastases. Two patients (6%) had not had enough information to classify them by condition. Ten patients were not trial-eligible; of these, one (10%) had a second cancer, one (10%) had received chemotherapy, and eight (80%) had low or non-rising prostate-specific antigen (PSA) levels. In the physician triage category, a physician determined that one patient (25%) needed immediate treatment, one had a job whose requirements ruled out participation, one was a patient for whom distance would be a problem (international patient), and one was not a good trial candidate. Eighty-nine percent of patients triaged had trials discussed and the remaining eleven percent were recommended a standard therapy or continuation of their current regimen. Nineteen of the 42 patients were interested when informed about clinical trials, and 23 (49%) were not interested. The reasons for lack of interest or willingness to participate were two-fold: distance was a problem for 19 patients (83%) and four preferred a specific treatment. Of those nineteen patients interested in trials, one (14%) was bound by actual and six (86%) by psychological distance of having a referring physician to return to. Of the patients who consented, one of the eleven (8%) failed the pre-enrollment screening protocol.

Drop-off between patient consultations and patient-trial fit was 47 patients; 70% (33/47) had no trial available, 21% were not eligible (10/47), and 9% (4/47) were not triaged. The drop-off to opportunity was five patients, which was made up entirely of patient’s lack of interest or doctor’s recommendation to continue with standard of care, and drop-off to acceptance was eight patients, made up of seven patients (88%) who did not consent and one (12%) who failed to meet the requirements of the study protocol.

### Patient subgroups

To illustrate how this framework might be used to track enrollment of patient subgroups, we show patients by race and ethnicity in Figure 1. Noting that this case series is based on a relatively small number, we do not report statistical significance. More than half of white patients dropped off over the patient-trial fit, while 20% of black patients dropped out at eligibility and at enrollment. The sole Hispanic patient was not interested in a trial.

## Discussion

Despite small numbers, this analytic, framework approach has given our cancer center a basis for troubleshooting clinical trial accrual for individuals of specific race or Hispanic ethnicity or other patient characteristics, although larger samples are necessary to assess this statistically. The approach has highlighted that trial availability and patient interest and consent are areas of potential improvement. To actually make improvements, more information may be necessary and a prospective, more real-time approach would be useful.

We believe our proposed order of transitions is endorsed by a number of ethical principles. Placing physician triage after availability and eligibility instead of before assures that the process is just. That is, subject eligibility is based on a systematic process utilizing objective criteria rather than relying on each physician’s ability to recall the criteria for each open trial for which a patient may be eligible. This approach also promotes the principle of beneficence by making sure that all who may benefit from enrollment are identified. In addition, our categorizing of the patients who were dropped out of consideration for a clinical trial also assures the process is just [51-53]. Moreover, this approach clarifies the basis for exclusion. No doubt additional reasons will be added to our list, and this may be accomplished with the help of the principles that guide any particular transition. Our emphasis on making certain that the trial is adequately described to the patient and discussed with the patient, signifies a respect for a patient’s right to choose and is the basis for shared information.

The proper first stage as described is establishing scientific oversight for a clinical trial enterprise. This stage is not to be underestimated in importance, but it is not generally a hurdle for a medical research institution that has established the necessary infrastructure to identify patients and maintain efficient approval of studies. Availability of clinical trials may be considered a measure of effectiveness at cancer centers, whose charge is to provide a broad portfolio of clinical trials for the patients it serves.

Seven transitions may be more than necessary and we have suggested a consolidation of transitional probabilities. These may be the key to standardization across studies, while reserving all seven for problem-solving within a study, a program or an institution. We think a systematic approach to therapeutic trials accrual will create uniform reporting and reduce physician triage of patients whom they judge ineligible, that is, unable to participate for various reasons. Investigators at other academic cancer centers have observed higher patient interest (84%) when physician perception of eligibility was implemented first, and that this combined with trial availability eliminated two thirds of all patients before interest was ascertained [13]. In a community-based cancer center, Go and colleagues [15] found the proportion of patients for whom a trial was available similar to the one observed in this pilot (66% as compared to 65%).

It is difficult to make comparisons across many studies, as standardized reporting and more importantly, standardized hierarchy of accrual to trials has not been available. In reporting to the NCI, standardization makes good sense, gives NCI cancer centers benchmarks, and highlights potential unique issues at each center. An issue highlighted by this analysis is distance from the center. One physician judged it an issue, nineteen patients cited it as a reason they were not interested, and one patient as the reason they could not consent. This totals 21 of the original 94 patients (22%) for whom there were trials; physicians thought they were good candidates and the patient was eligible, but distance was a problem, yet for different reasons that may have different remedies.

Again, this framework provides information for problem-solving and improving clinical trial participation by domain and by steps to accrual. Patient interest seemed to be a weak point in accrual but the primary reason for not participating might be totally understandable - distance from the cancer center (79% were not from Maryland). We would contrast this with 88% of accruals to trials being non-Maryland residents, so consideration of distance as a trial availability or eligibility criterion should be done with caution. Accruers may be those individuals who come to a regional cancer center in order to participate in trials [5].

## Conclusions

Examination of these transitions by domain points out that accrual to therapeutic trials may be enhanced with institutional/systemic attention to trial availability and eligibility, physician responsibilities to care for a patient and enroll them in clinical trials, and attention to patient interest and consent. In addition to distance, treatment preference is a potentially important reason for no interest in a trial, as is continuing care with their referring physician, for not consenting to a trial. These findings point out that trials closer to home or those with the involvement of the referring physicians may be the best solution. Reassuringly, this sampling of patients indicates the dedication of medical oncology clinical trialists to the steps of discussion and enrollment in trials. Still, this is just one cancer site and one treatment program.

Abstraction of patient flow periodically would aid in continuously tracking and troubleshooting clinical trial accrual. For instance, minimal eligibility criteria and requirements of clinical trial participation when possible, may promote more eligible candidates at the outset and consideration of a trial at ascertainment of patient interest. A continuous feedback loop of information for sustaining the pipeline of clinical trials for a broad spectrum of patients would be another possible aspect of data collection and analysis. To put these findings into action, we will design a trials data collection form/computer module that will organize screening for trials and facilitate discussion of trials and eligibility with new patients within the cancer program. This pilot study has provided sufficient information to apply this tool in our clinics.

While taking every patient through every step may entail some additional time, it assures that patient autonomy will be preserved and opportunity to participate will be maximized. Beyond patient interest these data point out what has been reported elsewhere: trial availability and eligibility are important considerations for improving accrual and access for many.

## Competing interests

The authors declare they have no competing interests.

## Authors’ contributions

Norma F. Kanarek originated the paper concept, reviewed the analyses, oversaw the chart abstraction, and was responsible for the initial writing of the manuscript. Marty S. Kanarek contributed to the use of ethical principles to the task of enrolling patients in clinical trials. Dare Olatoye conducted the chart review and initial analyses. Michael A. Carducci reviewed the collection of chart reviews and refereed the abstracted reasons for not participating in trials. All authors read and approved the final manuscript.

## References

[B1] Sateren WB Trimble EL Abrams J Brawley O Breen N Ford L McCabe M Kaplan R Smith M Ungerleider R Christian MC How sociodemographics, presence of oncology specialists, and hospital cancer programs affect accrual to cancer treatment trials J Clin Oncol 2002 20 2109 2117 10.1200/JCO.2002.08.056 11956272

[B2] Al-Refaie WB Vickers SM Zhong W Parsons H Rothenberger D Habermann EB Cancer trials versus the real world in the United States Ann Surg 2011 254 438 443 10.1097/SLA.0b013e31822a7047 21775882

[B3] Baquet CR Ellison GL Mishra SI Analysis of Maryland cancer patient participation in national cancer institute-supported cancer treatment clinical trials J Clin Oncol 2008 26 3380 3386 10.1200/JCO.2007.14.6027 18612153 PMC3602973

[B4] Tejeda HA Green SB Trimble EL Ford L High JL Ungerleider RS Friedman MA Brawley OW Representation of African-Americans, Hispanics, and whites in national cancer institute cancer treatment trials J Natl Cancer Inst 1996 88 812 816 10.1093/jnci/88.12.812 8637047

[B5] Kanarek NF Tsai HL Metzger-Gaud S Damron D Guseynova A Klamerus JF Rudin CM Geographic proximity and racial disparities in cancer clinical trial participation J Natl Compr Canc Netw 2010 8 1343 1351 21147901 10.6004/jnccn.2010.0102 PMC3201828

[B6] Onega T Duell EJ Shi X Wang D Demidenko E Goodman D Geographic access to cancer care in the U.S Cancer 2008 112 909 918 10.1002/cncr.23229 18189295

[B7] Su SC Kanarek N Fox MG Guseynova A Crow S Piantadosi S Spatial analyses identify the geographic source of patients at a National Cancer Institute Comprehensive Cancer Center Clin Cancer Res 2010 16 1065 1072 10.1158/1078-0432.CCR-09-1875 20103681

[B8] Martel CL Li Y Beckett L Chew H Christensen S Davies A Lam KS Lau DH Meyers FJ O'Donnell RT Richman C Scudder S Tanaka M Tuscano J Welborn J Wun T Gandara DR Lara PN Jr An evaluation of barriers to accrual in the era of legislation requiring insurance coverage of cancer clinical trial costs in California Cancer J 2004 10 294 300 10.1097/00130404-200409000-00006 15530258

[B9] Castel P Negrier S Boissel JP Why don't cancer patients enter clinical trials? A review. Eur J Cancer 2006 42 1744 1748 10.1016/j.ejca.2005.10.033 16777404

[B10] Grunfeld E Zitzelsberger L Coristine M Aspelund F Barriers and facilitators to enrollment in cancer clinical trials: qualitative study of the perspectives of clinical research associates Cancer 2002 95 1577 1583 10.1002/cncr.10862 12237928

[B11] Lara PN Jr Paterniti DA Chiechi C Turrell C Morain C Horan N Montell L Gonzalez J Davis S Umutyan A Martel CL Gandara DR Wun T Beckett LA Chen MS Jr Evaluation of factors affecting awareness of and willingness to participate in cancer clinical trials J Clin Oncol 2005 23 9282 9289 10.1200/JCO.2005.02.6245 16361626

[B12] Vickers AJ Kramer BS Baker SG Selecting patients for randomized trials: a systematic approach based on risk group Trials 2006 7 30 10.1186/1745-6215-7-30 17022818 PMC1609186

[B13] Lara PN Jr Higdon R Lim N Kwan K Tanaka M Lau DH Wun T Welborn J Meyers FJ Christensen S O’Donnell R Richman C Scudder SA Tuscano J Gandara DR Lam KS Prospective evaluation of cancer clinical trial accrual patterns: identifying potential barriers to enrollment J Clin Oncol 2001 19 1728 1733 11251003 10.1200/JCO.2001.19.6.1728

[B14] Schiller JH Study design issues and early stage non-small cell lung cancer Clin Cancer Res 2005 11 5030s 5032s 10.1158/1078-0432.CCR-05-9003 16000609

[B15] Go RS Frisby KA Lee JA Mathiason MA Meyer CM Ostern JL Walther SM Schroeder JE Meyer LA Umberger KE Clinical trial accrual among new cancer patients at a community-based cancer center Cancer 2006 106 426 433 10.1002/cncr.21597 16353206

[B16] Proctor JW Martz E Schenken LL Rainville R Marlowe U A screening tool to enhance clinical trial participation at a community center involved in a radiation oncology disparities program J Oncol Pract 2011 7 161 164 10.1200/JOP.2010.000135 21886496 PMC3092655

[B17] Altman DG Schulz KF Moher D Egger M Davidoff F Elbourne D Gotzsche PC Lang T The revised CONSORT statement for reporting randomized trials: explanation and elaboration Ann Intern Med 2001 134 663 694 11304107 10.7326/0003-4819-134-8-200104170-00012

[B18] Unger JM Coltman CA Jr Crowley JJ Hutchins LF Martino S Livingston RB Macdonald JS Blanke CD Gandara DR Crawford ED Albain KS Impact of the year 2000 Medicare policy change on older patient enrollment to cancer clinical trials J Clin Oncol 2006 24 141 144 10.1200/JCO.2005.02.8928 16330670

[B19] The National Commission for the Protection of Human Subjects of Biomedical and Behavioral Research The Belmont report: ethical principles and guidelines for the protection of human subjects of research 1979 Washington, DC: United States Department of Health, Education, and Welfare 25951677

[B20] Emanuel EJ Wendler D Grady C What makes clinical research ethical? JAMA 2000 283 2701 2711 10.1001/jama.283.20.2701 10819955

[B21] Brawley OW The study of accrual to clinical trials: can we learn from studying who enters our studies? J Clin Oncol 2004 22 2039 2040 10.1200/JCO.2004.02.926 15082727

[B22] Bubley GJ Carducci M Dahut W Dawson N Daliani D Eisenberger M Figg WD Freidlin B Halabi S Hudes G Hussain M Kaplan R Myers C Oh W Petrylak DP Reed E Roth B Sartor O Scher H Simons J Sinibaldi V Small EJ Smith MR Trump DL Vollmer R Wilding G Eligibility and response guidelines for phase II clinical trials in androgen-independent prostate cancer: recommendations from the Prostate-Specific Antigen Working Group J Clin Oncol 1999 17 3461 3467 10550143 10.1200/JCO.1999.17.11.3461

[B23] Scher HI Eisenberger M D'Amico AV Halabi S Small EJ Morris M Kattan MW Roach M Kantoff P Pienta KJ Carducci MA Agus D Slovin SF Heller G Kelly W Lange PH Petrylak D Berg W Higano C Wilding G Moul JW Partin AN Logothetis C Soule HR Eligibility and outcomes reporting guidelines for clinical trials for patients in the state of a rising prostate-specific antigen: recommendations from the Prostate-Specific Antigen Working Group J Clin Oncol 2004 22 537 556 14752077 10.1200/JCO.2004.07.099

[B24] Simon MS Du W Flaherty L Philip PA Lorusso P Miree C Smith D Brown DR Factors associated with breast cancer clinical trials participation and enrollment at a large academic medical center J Clin Oncol 2004 22 2046 2052 10.1200/JCO.2004.03.005 15082724

[B25] Behrendt CE Gehan EA Treatment-subgroup interaction: an example from a published, phase II clinical trial Contemp Clin Trials 2009 30 279 281 10.1016/j.cct.2009.02.002 19232549 PMC2732105

[B26] Kemeny MM Peterson BL Kornblith AB Muss HB Wheeler J Levine E Bartlett N Fleming G Cohen HJ Barriers to clinical trial participation by older women with breast cancer J Clin Oncol 2003 21 2268 2275 10.1200/JCO.2003.09.124 12805325

[B27] Sheldon JM Fetting JH Siminoff LA Offering the option of randomized clinical trials to cancer patients who overestimate their prognoses with standard therapies Cancer Invest 1993 11 57 62 10.3109/07357909309020261 8422596

[B28] Miller FG Rosenstein DL The therapeutic orientation to clinical trials N Engl J Med 2003 348 1383 1386 10.1056/NEJMsb030228 12672867

[B29] Mannel RS Walker JL Gould N Scribner DR Jr Kamelle S Tillmanns T McMeekin DS Gold MA Impact of individual physicians on enrollment of patients into clinical trials Am J Clin Oncol 2003 26 171 173 10.1097/00000421-200304000-00014 12714890

[B30] Meropol NJ Egleston BL Buzaglo JS Benson AB 3rd Cegala DJ Diefenbach MA Fleisher L Miller SM Sulmasy DP Weinfurt KP Cancer patient preferences for quality and length of life Cancer 2008 113 3459 3466 10.1002/cncr.23968 18988231 PMC2606934

[B31] Stiles CR Johnson L Whyte D Nergaard TH Gardner J Wu J Does increased patient awareness improve accrual into cancer-related clinical trials? Cancer Nurs 2011 34 E13 19 21252642 10.1097/NCC.0b013e31820254db

[B32] Shannon-Dorcy K Drevdahl DJ "I had already made Up My mind": patients and caregivers' perspectives on making the decision to participate in research at a US cancer referral center Cancer Nurs 2011 34 428 433 10.1097/NCC.0b013e318207cb03 21242765 PMC3134632

[B33] Weinfurt KP Castel LD Li Y Sulmasy DP Balshem AM Benson AB 3rd Burnett CB Gaskin DJ Marshall JL Slater EF Schulman KA Meropol NJ The correlation between patient characteristics and expectations of benefit from phase I clinical trials Cancer 2003 98 166 175 10.1002/cncr.11483 12833469

[B34] Biedrzycki BA Factors and outcomes of decision making for cancer clinical trial participation Oncol Nurs Forum 2011 38 542 552 10.1188/11.ONF.542-552 21875841

[B35] Brown RF Butow PN Butt DG Moore AR Tattersall MH Developing ethical strategies to assist oncologists in seeking informed consent to cancer clinical trials Soc Sci Med 2004 58 379 390 10.1016/S0277-9536(03)00204-1 14604623

[B36] Kass N Taylor H Fogarty L Sugarman J Goodman SN Goodwin-Landher A Carducci M Hurwitz H Purpose and benefits of early phase cancer trials: what do oncologists say? what do patients hear? J Empir Res Hum Res Ethics 2008 3 57 68 19385771 10.1525/jer.2008.3.3.57 PMC2861824

[B37] Kass NE Sugarman J Medley AM Fogarty LA Taylor HA Daugherty CK Emerson MR Goodman SN Hlubocky FJ Hurwitz HI Carducci M Goodwin-Landher A An intervention to improve cancer patients' understanding of early-phase clinical trials IRB 2009 31 1 10 19552233 PMC2872090

[B38] Ruckdeschel JC Albrecht TL Blanchard C Hemmick RM Communication, accrual to clinical trials, and the physician-patient relationship: implications for training programs J Cancer Educ 1996 11 73 79 8793646 10.1080/08858199609528399

[B39] Meropol NJ Buzaglo JS Millard J Damjanov N Miller SM Ridgway C Ross EA Sprandio JD Watts P Barriers to clinical trial participation as perceived by oncologists and patients J Natl Compr Canc Netw 2007 5 655 664 17927923 10.6004/jnccn.2007.0067

[B40] Sulmasy DP Astrow AB He MK Seils DM Meropol NJ Micco E Weinfurt KP The culture of faith and hope: patients' justifications for their high estimations of expected therapeutic benefit when enrolling in early phase oncology trials Cancer 2010 116 3702 3711 10.1002/cncr.25201 20564120 PMC3644988

[B41] Rasco DW Xie Y Yan J Sayne JR Skinner CS Dowell JE Gerber DE The impact of consenter characteristics and experience on patient interest in clinical research Oncologist 2009 14 468 475 10.1634/theoncologist.2008-0268 19401521

[B42] Lee MM Chamberlain RM Catchatourian R Hiang J Kopnick M Ray P Vijayakumar S Social factors affecting interest in participating in a prostate cancer chemoprevention trial J Cancer Educ 1999 14 88 92 10397483 10.1080/08858199909528586

[B43] Sabesan S Burgher B Buettner P Piliouras P Otty Z Varma S Thaker D Attitudes, knowledge and barriers to participation in cancer clinical trials among rural and remote patients Asia Pac J Clin Oncol 2011 7 27 33 10.1111/j.1743-7563.2010.01342.x 21332648

[B44] Stepan KA Gonzalez AP Dorsey VS Frye DK Pyle ND Smith RF Throckmorton TA Villejo LA Cantor SB Recommendations for enhancing clinical trials education: a review of the literature J Cancer Educ 2011 26 64 71 10.1007/s13187-010-0160-4 20862574

[B45] Daugherty CK Impact of therapeutic research on informed consent and the ethics of clinical trials: a medical oncology perspective J Clin Oncol 1999 17 1601 1617 10334550 10.1200/JCO.1999.17.5.1601

[B46] Mulhall JP Montorsi F Evaluating preference trials of oral phosphodiesterase 5 inhibitors for erectile dysfunction Eur Urol 2006 49 30 37 10.1016/j.eururo.2005.09.001 16263207

[B47] Wolf AM Nasser JF Schorling JB The impact of informed consent on patient interest in prostate-specific antigen screening Arch Intern Med 1996 156 1333 1336 10.1001/archinte.1996.00440110105014 8651843

[B48] Miller FG Joffe S Balancing access and evaluation in the approval of new cancer drugs JAMA 2011 305 2345 2346 10.1001/jama.2011.784 21642688

[B49] Agrawal M Grady C Fairclough DL Meropol NJ Maynard K Emanuel EJ Patients' decision-making process regarding participation in phase I oncology research J Clin Oncol 2006 24 4479 4484 10.1200/JCO.2006.06.0269 16983117

[B50] Klamerus JF Bruinooge SS Ye X Klamerus ML Damron D Lansey D Lowery JC Diaz LA Jr Ford JG Kanarek N Rudin CM The impact of insurance on access to cancer clinical trials at a comprehensive cancer center Clin Cancer Res 2010 16 5997 6003 10.1158/1078-0432.CCR-10-1451 21169253 PMC3715082

[B51] Mills EJ Seely D Rachlis B Griffith L Wu P Wilson K Ellis P Wright JR Barriers to participation in clinical trials of cancer: a meta-analysis and systematic review of patient-reported factors Lancet Oncol 2006 7 141 148 10.1016/S1470-2045(06)70576-9 16455478

[B52] Schutta KM Burnett CB Factors that influence a patient's decision to participate in a phase I cancer clinical trial Oncol Nurs Forum 2000 27 1435 1438 11058975

[B53] Wright JR Crooks D Ellis PM Mings D Whelan TJ Factors that influence the recruitment of patients to phase III studies in oncology: the perspective of the clinical research associate Cancer 2002 95 1584 1591 10.1002/cncr.10864 12237929

